# Supplementary material for: Unsupervised detection of novel SARS-CoV-2 mutations and lineages in wastewater samples using long-read sequencing
Source: BMC Genomics. 2025 Jan 29;26:87. doi: 10.1186/s12864-025-11255-z (PMC11780762; doi:10.1186/s12864-025-11255-z)
Supplement: Supplementary file 2 — Supplementary Material 2. [file 12864_2025_11255_MOESM2_ESM.docx]

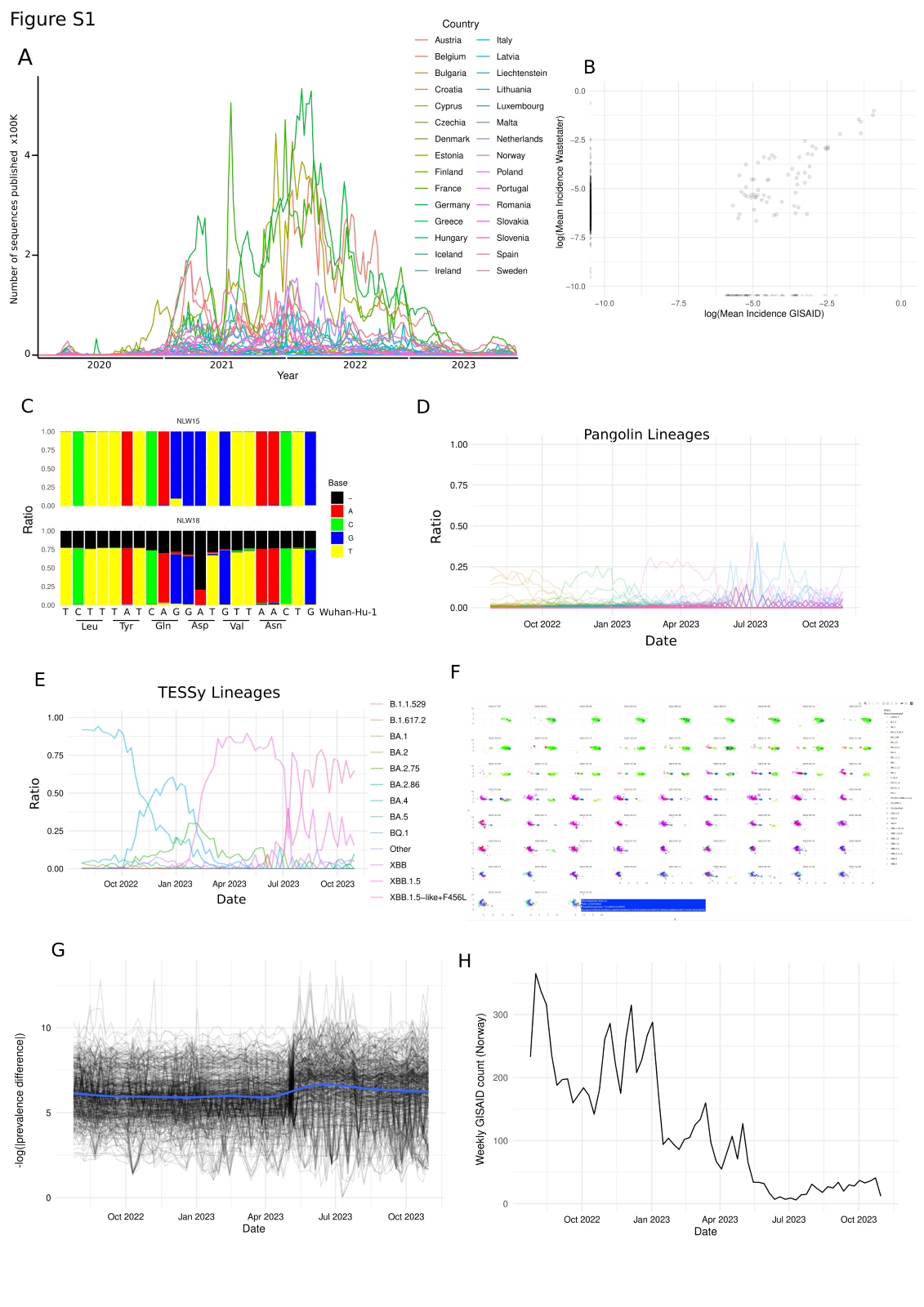


**Figure S1: Data controls. A.** Line plot showing the number of sequences from clinical samples deposited in GISAID by the different European countries. The countries are represented with different colors. **B.** Scatter plot showing the correlation between the average incidence of the *mutation-lineages* found in clinical samples and wastewater. Each point represents a combination of mutations (i.e., a *mutation-lineage*). The points aligned on the bottom or left borders represent *mutation-lineages* not found on wastewater and GISAID respectively. **C.** Bar plots showing the proportion of the different nucleotides mapped on the 614^th^ codon area for NWL15 and NWL18 lineages. The nucleotide and amino acid sequences of the Wuhan-Hu-1 reference are shown. The different colors show the different nucleotides (black shows that a deletion was mapped). **D.** Line plot representing the temporal incidence of the different Pangolin lineages in Norway. Each color represents a different lineage. **E.** Line plot representing the temporal incidence of the different TESSy lineages in Norway. Each color represents a different lineage. **F.** Screenshot of the 2D-map HTML widget generated by HERCULES. **G.** Line plot showing the variability in the absolute differences between the detected point mutations (See Fig. 4B). Each line represents a point mutation. The blue line represents the smoothed average of the lines. **H.** Line plot showing the number of weekly Norwegian clinical samples deposited in GISAID.


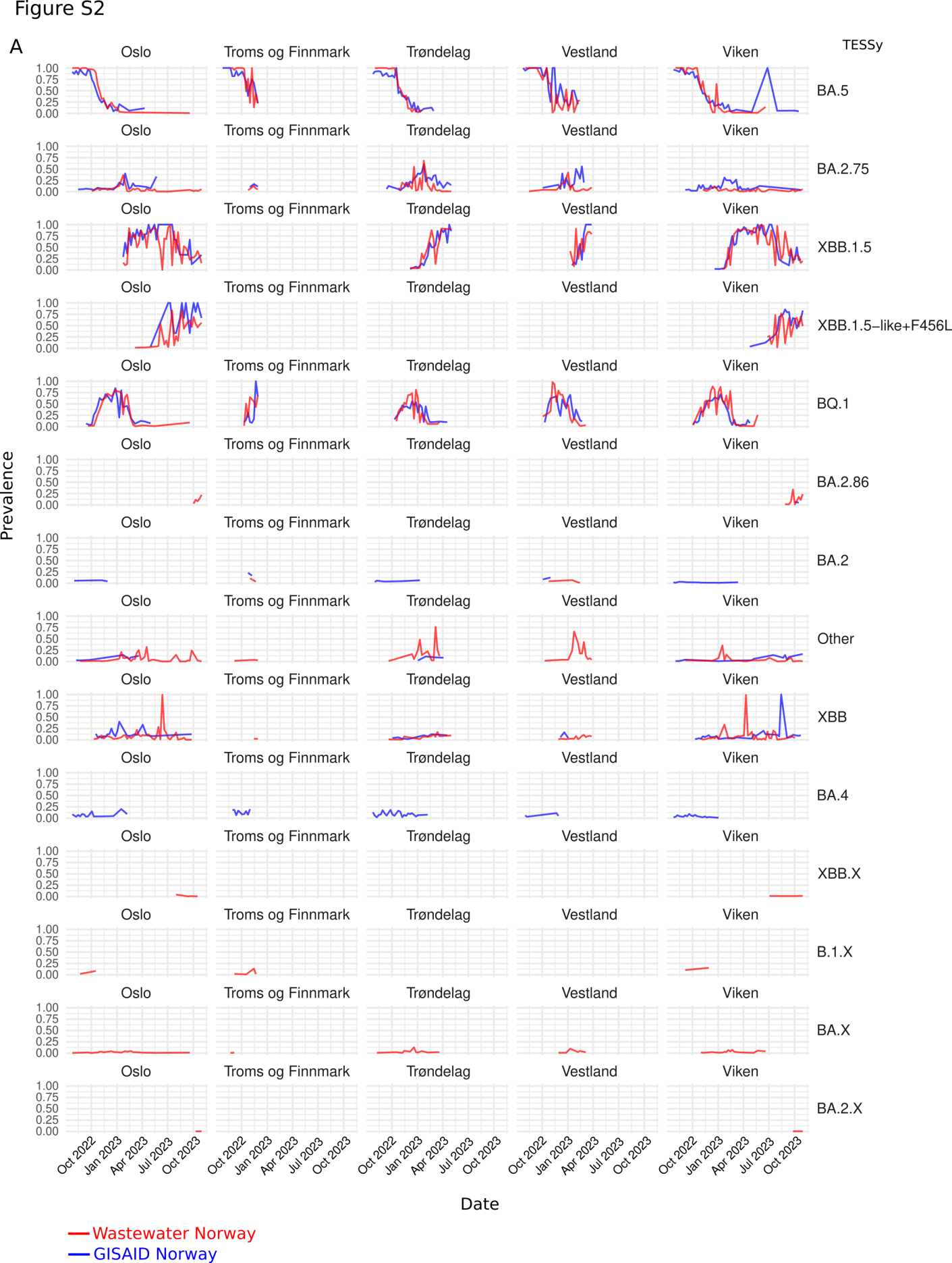


**Figure S2: County-wise identification of SARS-CoV-2 TESSy lineages in wastewater using HERCULES. A.** Line plots of the temporal prevalence of The European Surveillance System (TESSy) lineages present in wastewater (red lines) and clinical data from GISAID (blue lines) aggregated by county. Only the clinical data from Oslo and Viken are shown after May 2023 due to the absence of wastewater samples from the rest of counties.


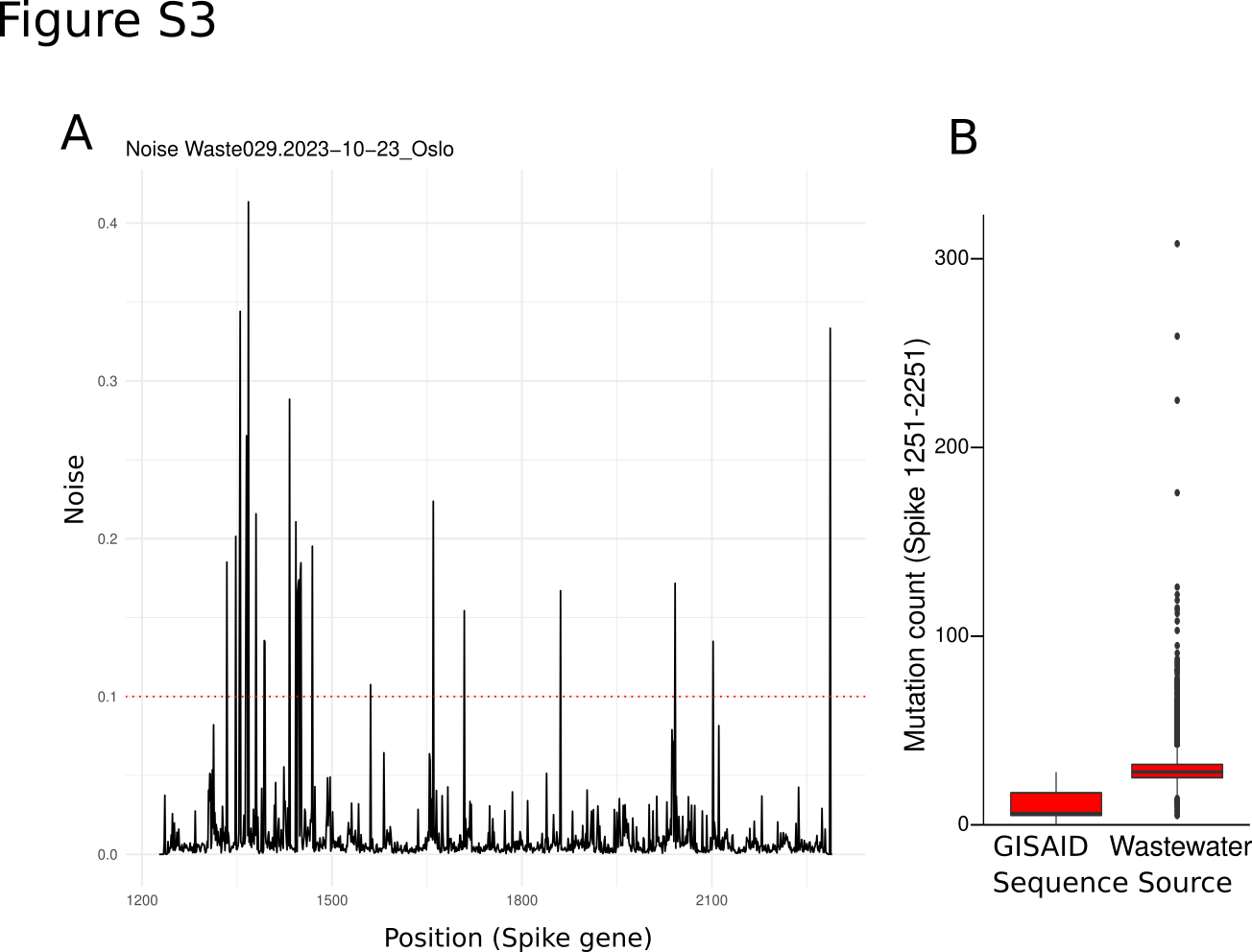


**Figure S3: Controls used during HERCULES development. A.** Representative noise plot generated by HERCULES at running time. On the X-axis the nucleotide position regarding the Spike gene is shown. On the Y-axis the noise (i.e., the ratio of reads that have a nucleotide different from the most common nucleotide found in a particular position). The dashed red line represents the cutoff used by HERCULES to find positions of interest. **B.** Box plots showing the number of mutations at nucleotide level between the positions 1251 and 2251 of the Spike gene in clinical samples (GISAID) and wastewater.
